# Taxonomical study on the newly-recorded genus *Falsonnannocerus* Pic from China (Coleoptera, Tenebrionidae, Stenochiinae)

**DOI:** 10.3897/BDJ.9.e73232

**Published:** 2021-12-01

**Authors:** Jiang Zhu, Cheng-Bin Wang, Bao-Ying Feng

**Affiliations:** 1 Conature Company Limited, 12 Dashatou Second Road, Guangzhou, China Conature Company Limited, 12 Dashatou Second Road Guangzhou China; 2 Engineering Research Center for Forest and Grassland Disaster Prevention and Reduction, Mianyang Normal University, 166 Mianxing West Road, Mianyang, China Engineering Research Center for Forest and Grassland Disaster Prevention and Reduction, Mianyang Normal University, 166 Mianxing West Road Mianyang China; 3 Guangzhou Haizhu Wetland Center for Research and Education, 168 Xinjiao Middle Road, Guangzhou, China Guangzhou Haizhu Wetland Center for Research and Education, 168 Xinjiao Middle Road Guangzhou China

**Keywords:** darkling beetle, Cnodalonini, new species, newly-recorded species, taxonomy, Oriental Region

## Abstract

**Background:**

The genus *Falsonannocerus* Pic, 1947 (Coleoptera, Tenebrionidae, Stenochiinae, Cnodalonini) includes 13 known species occurring in West Africa, South Asia and Southeast Asia. The bionomics of species in this genus are unknown.

**New information:**

The genus *Falsonnannocerus* Pic, 1947 is recorded for the first time from China, including two species: *F.thailandicus* Masumoto, 1986 from Yunnan and *F.haizhuensis* sp. n. from Guangdong. Morphological characters of the two species are illustrated and compared. The photographs of female reproductive system, which were never shown for this genus before, are also presented.

## Introduction

The genus *Falsonannocerus* Pic, 1947 belongs to the tribe Cnodalonini (Coleoptera, Tenebrionidae, Stenochiinae) and was described by Pic (1947), based on a single species *Falsonannocerusdentaticeps* Pic, 1947 from Côte d'Ivoire, which was fixed as the type species of the genus by monotypy. Subsequently, 12 species, assigned to the genus *Falsonannocerus*, were described: one species from Sri Lanka (Kaszab 1980a) and 11 species from Southeast Asia (Kaszab 1980b, Masumoto 1985, Masumoto 1986, Masumoto 1998, Grimm 2013).

In the present paper, we describe or re-describe two *Falsonannocerus* species: *F.thailandicus* Masumoto, 1986 from Yunnan Province, southwest China and *F.haizhuensis* sp. n. from Guangdong Province, southeast China, representing the first record of the genus from China. Important distinguishing morphological characters of the two species are illustrated with colour plates. The female reproductive system of the genus is provided.

## Materials and methods

Specimens were relaxed and softened in hot water for 24 hours, then transferred to distilled water to clean, observe and dissect. In order to examine the genitalia of both sexes, the abdomens were detached and treated with a 10% solution of potassium hydroxide (KOH) for 12 hours, then transferred to distilled water to flush the remaining KOH and stop any further bleaching. After examination, the body parts were mounted on a glass slide with Euparal Mounting Medium for future studies. Photographs of the habitus were taken using microlens on a Canon 5D IV. Detailed photographs with flashlight were performed using a Olympus 10X microlens with a Canon 5D IV. The final deep focus images were created with Zerene Stacker 1.04 stacking software. Adobe Photoshop CS6 was used for post-processing. The terminology adopted in the paper for ovipositor and female genital tubes follows Tschinkel and Doyen (1980).

The material examined for this study is deposited in the following private and public collections: **CJZG**: collection of Jiang Zhu, Guangzhou, Guangdong, China; **CDYZ**: collection of Deyao Zhou, Shanghai, China; **MYNU**: insect collection of Mianyang Normal University, Mianyang, China; **NSMT**: National Science Museum, Tsukuba, Japan.

Measurement criteria in millimetres (mm) are as follows: **antennal length**: length between the base of scape and the apex of ultimate antennomere; **body length**: length between the apices of mandibles and the elytral apices along the mid-line; **elytral length**: length between the basal border and the apex of elytra along suture; **elytral width**: widest part of both elytra combined; **head length**: length between the anterior margin of epistoma and the anterior margin of pronotum along the mid-line; **head width**: widest part of head (including eyes); **pronotal length**: length of the pronotum along the mid-line; **pronotal width**: widest part of pronotum.

## Taxon treatments

### Falsonannocerus thailandicus

Masumoto, 1986

3360B37C-272C-563B-B6B5-9C0614B39F38

#### Materials

- **Type status:** Holotype. **Occurrence:** recordedBy: S. Fukuda; individualCount: 1; sex: male; **Location:** country: Thailand; verbatimLocality: Chiang Dao; **Identification:** identifiedBy: K. Masumoto; **Event:** year: 1980; month: 5; day: 2; **Record Level:** institutionCode: NSMT
- **Type status:** Other material. **Occurrence:** recordedBy: Gui-Chang Liu; individualCount: 3; sex: 2 males, 1 female; **Location:** country: China; stateProvince: Yunnan; verbatimLocality: Yingjiang County, Nongzhang Town [盈江县，弄璋镇]; **Event:** year: 2021; month: 4; **Record Level:** collectionCode: CDYZ

#### Description

**Male.** Body 8.7 mm in length, 3 times as long as wide, widest slightly behind middle of elytra. Lengths of body parts (mm): head (1.2), eye (0.3), antenna (1.8), pronotum (1.7), elytra (5.8); width: head (1.4), eye (0.3), pronotum (1.7), elytra (2.6).

**Figure 1.**
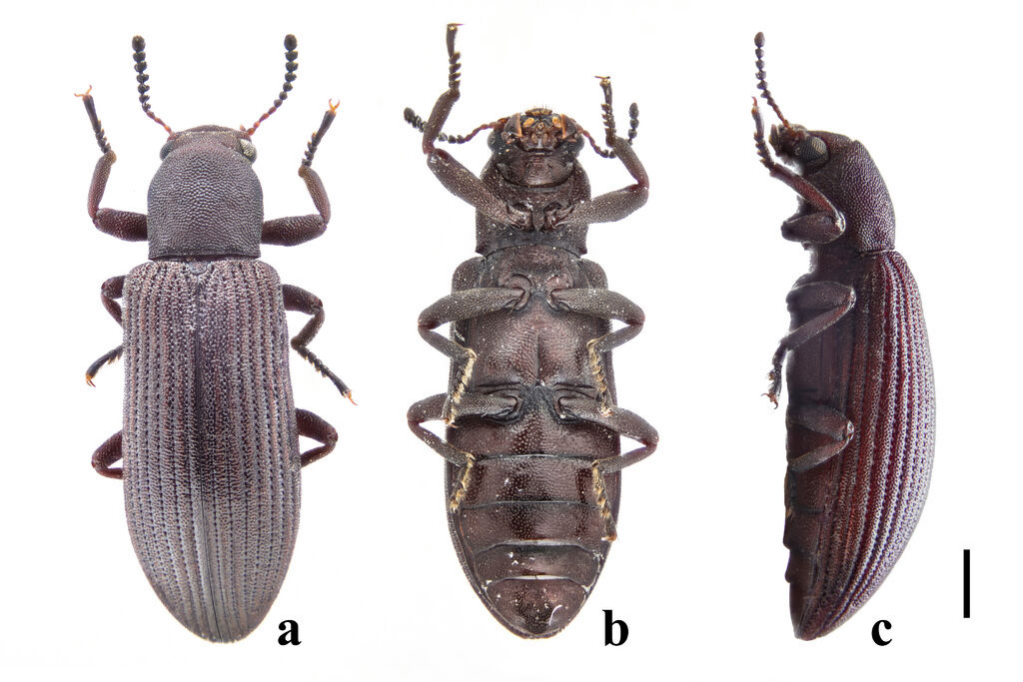
Habitus of *Falsonannocerusthailandicus* Masumoto, 1986 from Yunnan, China. Scale bar = 1 mm. **a**: dorsal view; **b**: ventral view; **c**: lateral view.

Habitus (Figs 1, 2a). Body oblong, slender, moderately convex dorsally and feebly convex ventrally, lustrous. Colour mostly reddish-brown; eyes, apical seven antennomeres and tarsi blackish. Body mostly covered with short, thick, yellowish setae; gula mostly glabrous; antennae, apices of tibiae and tarsi covered with longer and thinner setae; labrum with longer and stronger setae at outer margin.

Head transversely elliptical, widest at eye level, strongly convex posteriorly, almost wholly covered with dense and coarse punctures. Epistoma rather small, crescent. Genae oblique, with outer margins rounded. Eyes large, convex laterally, with strong inner ocular sulci. Gula (Fig. 3a) convex, strongly wrinkled.

Mouthparts. Labrum liguliform, surface microreticulate. Maxillary palpi with terminal palpomere securiform. Labial palpi with terminal palpomere elongate, conical. Mentum hippocrepiform, with 6 setae. Submental peduncle trapezoidal.

Antennae (Fig. 4a) short and robust, strongly flattened, about 1/4 length of body and 1.2 times as long as head width. Length ratio of antennomeres from base to apex: 1.2:1.1:1.5:1.2:1.2:1.0:1.2:1.2:1.1:1.2:2.0; width ratio: 1.3:1.0:1.1:1.0:1.2:1.6:1.8:2.2:2.3:2.3:2.3. Scape robust, 1.3 times as long as wide; pedicel to antennomere III subcylindrical, without stalks; antennomere V 1.4 times as wide as long, moderately dilated; VI–X dilated, somewhat cyathiform, with short stalks; XI longitudinally ovate; apical six antennomeres forming an oblong club, 1.5 times as long as basal four.

Prothorax. Pronotum (Fig. 3c) subcylindrical, length equal to width, broadest at anterior 2/5. Margins not beaded; anterior margin arched; basal margin slightly emarginate laterally; lateral margins feebly crenulate, weakly emarginate near base. Anterior corners subrectangular, with apices rounded in dorsal view; posterior corners slightly projected, acute. Disc moderately convex; surface covered with dense, well-defined, coarse, deep and subround punctures, each with a seta in middle; intervals distinctly carinate. Surface along basal margin with a shallow transverse furrow. Pronotal hypomera punctate exactly as pronotum. Prosternum transverse, much more sparsely punctate; intervals microreticulate. Prosternal process (Fig. 3e) narrow, linguiform, slightly elevated between coxae, apex reaching posterior margin of procoxae.

Scutellar shield linguiform, rounded at apex. Disc densely and minutely tuberculate.

Elytra elongate, 2.2 times as long as widest part, widest at apical 3/7. Elytra strongly convex in lateral view, especially in apical half. Lateral margins gradually widened from humeri to apical 3/7, then gradually narrowing to rounded apices. Each elytron with nine irregular rows of close and coarse punctures and short scutellary row in basal 1/6; intervals feebly convex and densely covered with small tubercles throughout. Epipleura wide at base, narrowing towards apex and terminating near apex and sparsely and finely punctate. Hind wings fully developed. Mesoventrite weakly convex towards middle, sparsely punctate, denser posteriorly. Mesepisternum and mesepimeron both triangular, densely punctate. Metaventrite densely punctate and finely grooved along mid-line in posterior half. Metepisternum rather long and thin, densely punctate.

Legs. Femora weakly dilated. Tibiae straight, more or less clavate; protibiae slightly bent near apex of lower side. Tarsi stout. Femora and tibiae densely and coarsely punctate. Setae longer and denser in lower sides of all legs.

**Figure 6.**
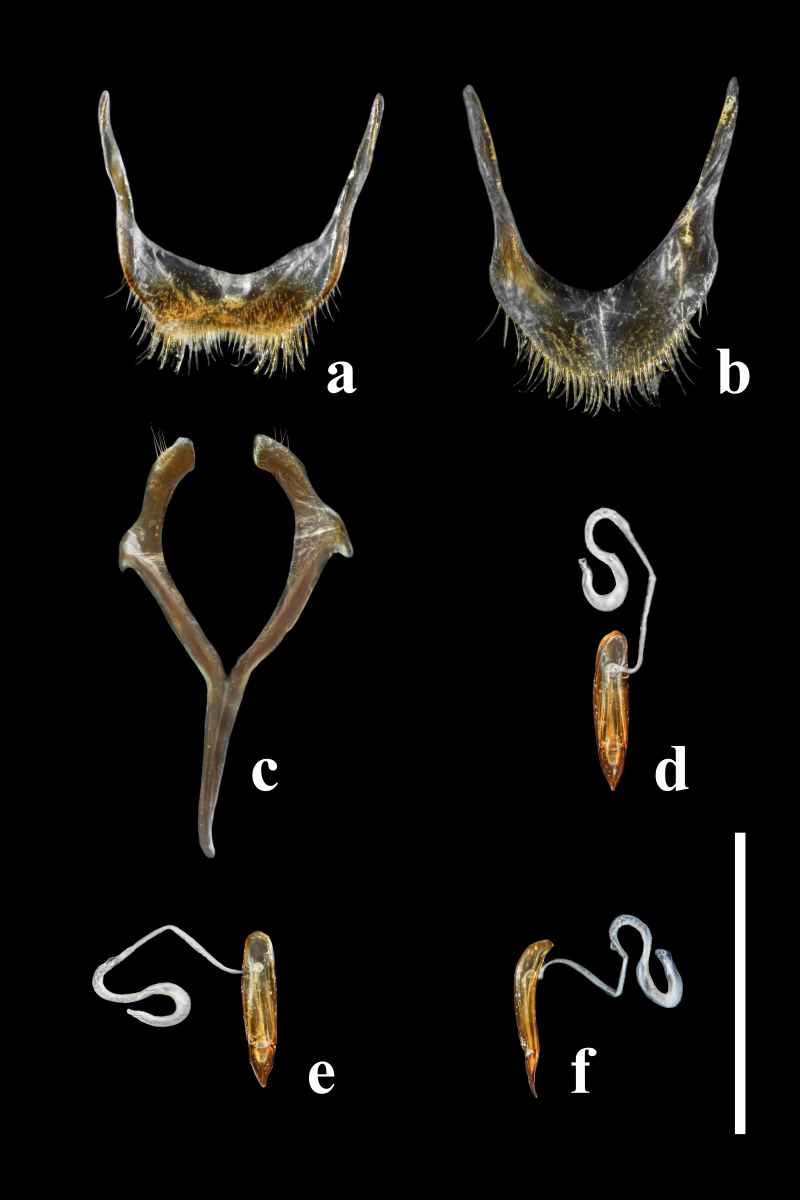
*Falsonannocerusthailandicus* Masumoto, 1986, male. Scale bars = 1 mm. **a**: abdominal sternite VIII; **b**: abdominal tergite VIII; **c**: male spiculum gastrale; **d**: aedeagus in ventral view; **e**: aedeagus in dorsal view; **f**: aedeagus in lateral view.

Abdomen. Abdominal sternites III–VI transverse, almost equal in length, densely punctate; intercoxal process on sternite III large, subtriangular; sternite VII semicircular, widely rounded at posterior margin; sternite VIII (Fig. 6a) widely emarginate at posterior margin, transversely membranous in anterior half. Spiculum gastrale (Fig. 6c) with branches about 1.3 times as long as stem. Abdominal tergite VII (Fig. 5a) semicircular, widely rounded at posterior margin; tergite VIII (Fig. 6b) rounded at posterior margin, with subtriangular membranous area along mid-line and two oblique membranous areas laterally.

**Figure 7.**
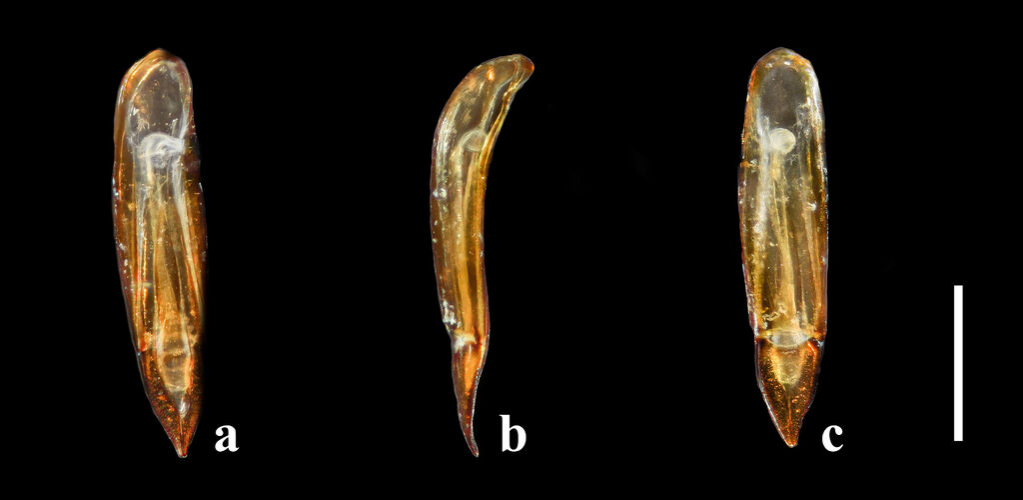
Aedeagus of *Falsonannocerusthailandicus* Masumoto, 1986 from Yunnan, China. Scale bar = 0.2 mm. **a**: dorsal view; **b**: ventral view; **c**: lateral view.

Aedeagus (Fig. 6d–f and Fig. 7) slender, simple, 0.52 mm in length and 0.09 mm in width. Basal piece subovate, 0.41 mm in length, widest at about basal 2/5; weakly curved in lateral view. Apical piece elongate subtriangular, thickened at apex, 0.11 mm in length; thick, weakly curved in lateral view.

**Figure 8.**
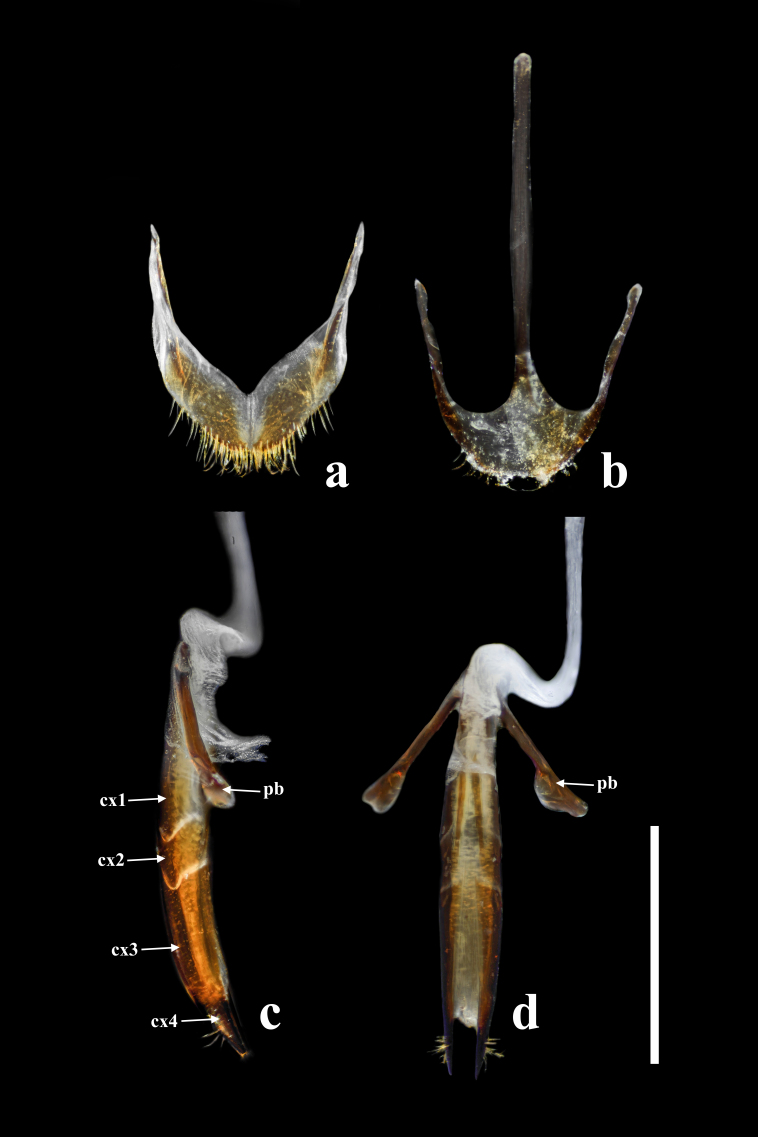
*Falsonannocerusthailandicus* Masumoto, 1986, female. Scale bars = 1 mm. Abbreviations: cx1–cx4, coxite lobes 1–4; pb, paraproct baculus. **a**: abdominal tergite VIII; **b**: abdominal sternite VIII; **c**: ovipositor in lateral view; **d**: ovipositor in dorsal view.

**Female.** Almost same as male in general appearance. Abdominal tergite VII (Fig. 5b) more curved at posterior margin; tergite VIII (Fig. 8a) rounded at posterior margin, with wide longitudinal membranous area along mid-line. Abdominal sternite VIII (Fig. 8b) rounded at posterior margin, disc largely membranous; spiculum ventrale rather long and slender. Ovipositor (Fig. 8c, d) strongly sclerotised, gently curved dorsally, with coxites slightly fused; coxite lobe 1 the longest, length ratio of coxite lobes 1–4 about 3:1:2:1; paraproct baculus enlarged at apex. Female genital tubes (Fig. 9a): vagina rather slender in anterior part, adruptly inflated in fusiform posterior part; oviduct produced from posterior part of vagina; spermathecal duct long, about 1.7 times as long as ovipositor, opening at anterior end of vagina; spermatheca globular, at apex of spermathecal duct; bursa copulatrix and spermathecal accessory gland absent.

#### Distribution

China (Yunnan), Thailand.

### Falsonannocerus haizhuensis

Zhu, Wang & Feng sp. n.

91E97935-0836-5D19-8AA8-38BFE2C2713C

69C9E35F-46DD-4A93-97E9-F69FE98C6EA5

#### Materials

- **Type status:** Holotype. **Occurrence:** recordedBy: Jiang Zhu & Wen-Ting Chen; individualCount: 1; sex: female; **Location:** country: China; stateProvince: Guangdong; verbatimLocality: Guangzhou, Haizhu National Wetland Park [海珠国家湿地公园]; verbatimElevation: 8 m; **Event:** year: 2021; month: 6; day: 9; **Record Level:** institutionCode: MYNU

#### Description

**Holotype female.** Body 6.2 mm in length, 2.5 times as long as wide, widest slightly behind middle of elytra. Lengths of body parts (mm): head (0.9), eye (0.3), antenna (1.2), pronotum (1.2), elytra (4.1); width: head (1.1), eye (0.2), pronotum (1.2), elytra (1.8).

**Figure 10.**
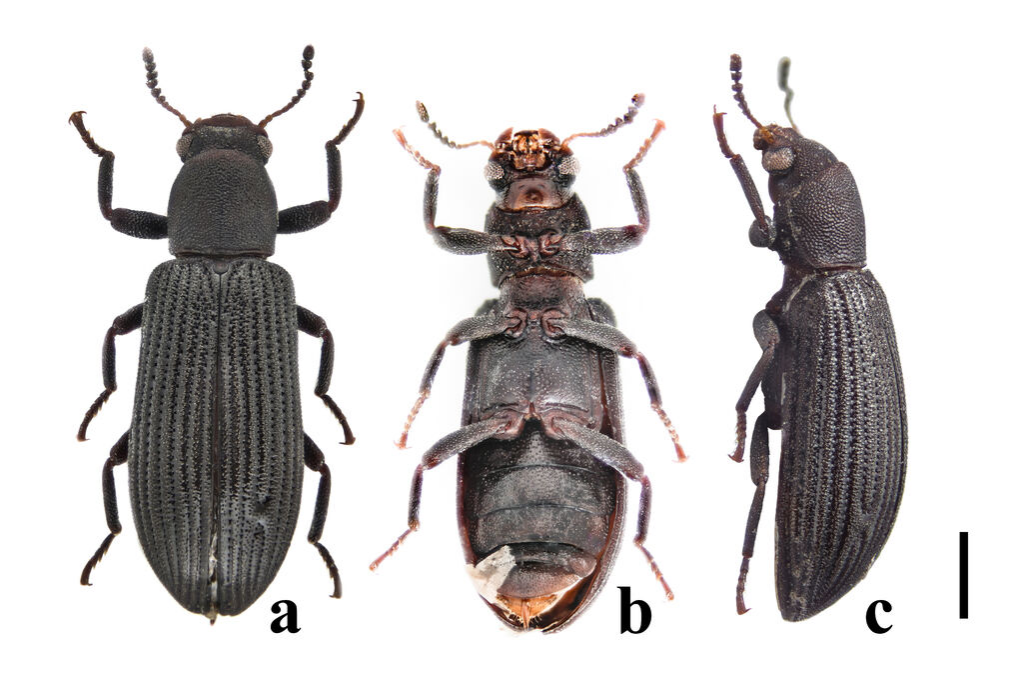
Habitus of *Falsonannocerushaizhuensis* sp. n. from Guangdong, China. Scale bar = 1 mm. **a**: dorsal view; **b**: ventral view; **c**: lateral view.

Habitus (Fig. 10). Body oblong, slender, moderately convex dorsally and feebly convex ventrally, dull. Colour mostly blackish; mouthparts, gula, basal four antennomeres and tarsi dark brownish. Body mostly covered with short, thick, yellowish setae; gula mostly glabrous; antennae, apices of tibiae and tarsi covered with longer and thinner setae; labrum with longer and stronger setae at outer margin.

Head transversely elliptical, widest at eye level, strongly convex posteriorly, almost wholly covered with dense and coarse punctures. Epistoma rather small, crescentic. Genae oblique, with outer margins rounded. Eyes large, convex laterally, with strong inner ocular sulci. Gula (Fig. 3b) convex, almost smooth.

Mouthparts. Labrum liguliform, surface microreticulate. Maxillary palpi with terminal palpomere securiform. Labial palpi with terminal palpomere elongated conical. Mentum hippocrepiform, with 3–4 setae. Submental peduncle trapezoidal.

Antennae (Fig. 4b) short and robust, strongly flattened, about 1/4 length of body and as long as head width. Length ratio of antennomeres from base to apex: 2.2:1.7:2.0:1.3:1.0:1.5:1.8:1.8:1.5:2.0:3.3; width ratio: 1.3:1.3:1.0:1.0:1.3:1.8:2.2:2.7:2.0:2.2:2.3. Scape robust, 1.6 times as long as wide; pedicel to antennomere V subcylindrical, without stalks; antennomere V 1.3 times as long as wide, moderately dilated; VI–X dilated, somewhat cyathiform, with short stalks; XI longitudinally ovate; apical six antennomeres forming an oblong club, 1.7 times as long as basal four.

Prothorax. Pronotum (Fig. 3d) subcylindrical, length equal to width, broadest at middle. Margins not beaded; anterior margin arched, slightly straight in middle; basal margin slightly emarginate laterally; lateral margins feebly crenulate, slightly emarginate near base. Anterior corners subrectangular, with apices rounded in dorsal view; posterior corners obtusely rounded. Disc moderately convex; surface covered with dense, ill-defined, coarse, shallow and subround punctures, each with a seta in middle; intervals vaguely carinate. Surface along basal margin with a moderate deep transverse groove. Pronotal hypomera punctate exactly as pronotum. Prosternum transverse, much more sparsely punctate; intervals microreticulate. Prosternal process (Fig. 3f) narrow, linguiform, slightly elevated between coxae, apex reaching posterior margin of prothorax.

Scutellar shield linguiform, rounded at apex. Disc densely and minutely wrinkled.

Elytra elongate, 2.3 times as long as widest part, widest at apical 3/7. Elytra strongly convex in lateral view, especially in apical half. Lateral margins gradually widened from humeri to apical 3/7, then gradually narrowing to rounded apices. Each elytron with nine irregular rows of close and coarse punctures and short scutellary row in basal 1/6; intervals feebly convex and sparsely covered with small tubercles throughout. Epipleura wide at base, narrowing towards apex and terminating near apex and sparsely and finely punctate. Hind wings fully developed. Mesoventrite weakly convex towards middle, sparsely punctate, denser posteriorly. Mesepisternum and mesepimeron both triangular, densely punctate. Metaventrite densely punctate and finely grooved along mid-line in posterior half. Metepisternum rather long and thin, densely punctate.

Legs. Femora weakly dilated. Tibiae straight, more or less clavate; protibiae slightly bent near apex of lower side. Tarsi stout. Femora and tibiae densely and coarsely punctate. Setae longer and denser in lower sides of all legs.

**Figure 11.**
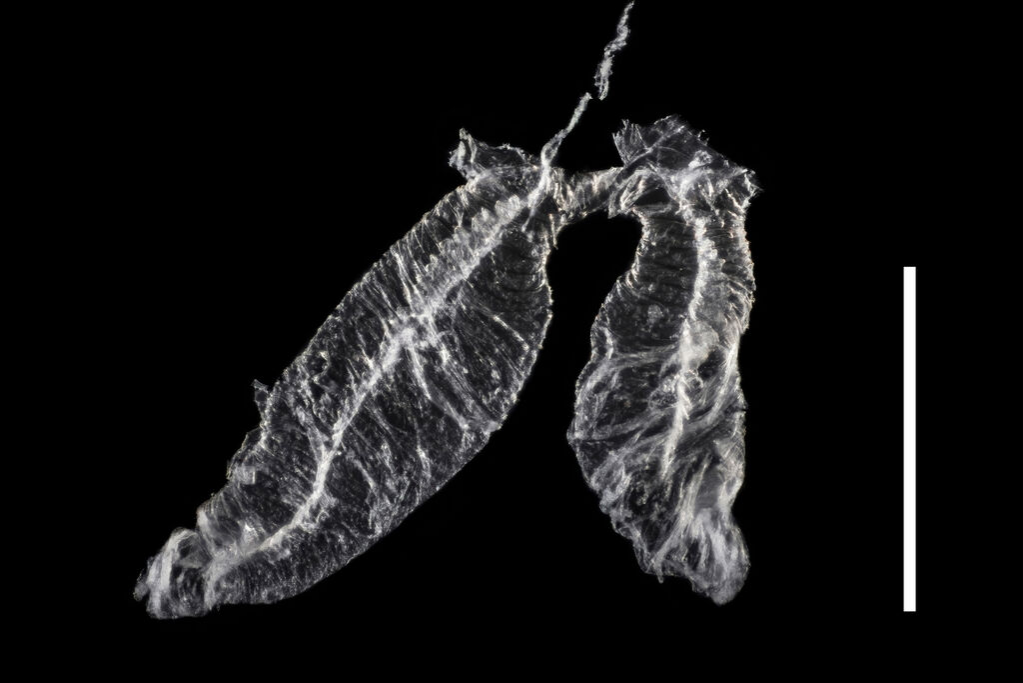
Defensive glands of *Falsonannocerushaizhuensis* sp. n. Scale bars = 1 mm.

**Figure 12.**
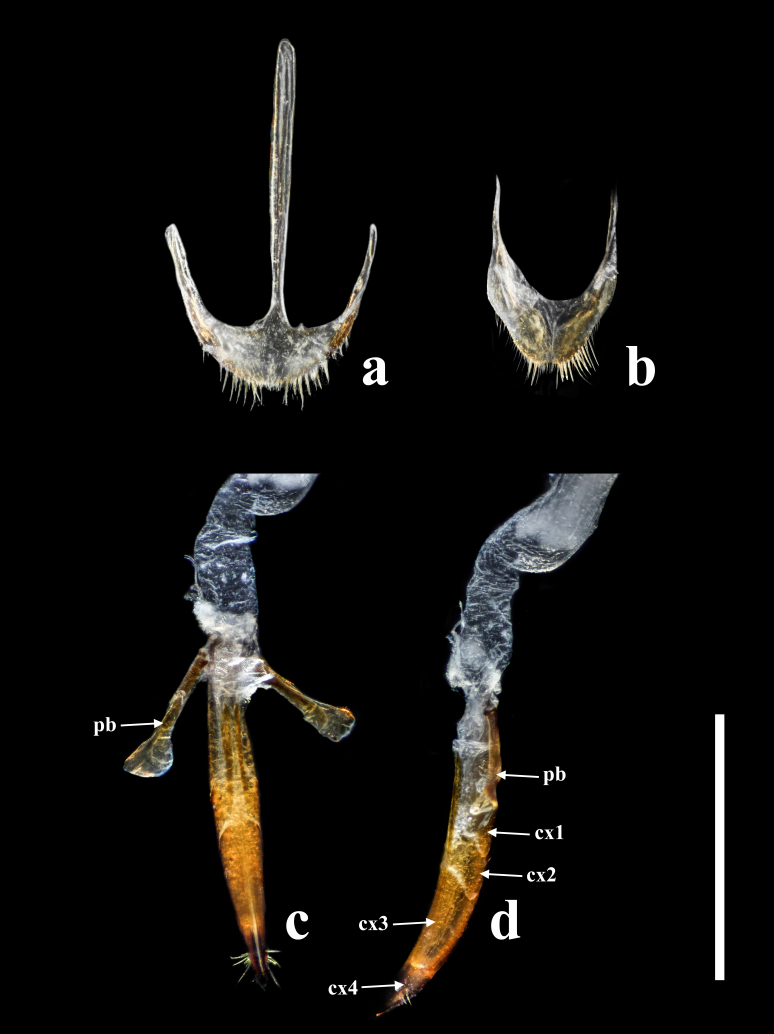
*Falsonannocerushaizhuensis* sp. n., female. Scale bars = 1 mm. Abbreviations: cx1–cx4, coxite lobes 1–4; pb, paraproct baculus. **a**: abdominal sternite VIII; **b**: abdominal tergite VIII; **c**: ovipositor in dorsal view; **d**: ovipositor in lateral view.

Abdomen. Abdominal sternites III–VI transverse, almost equal in length, densely punctate; intercoxal process on sternite III large, subtriangular; sternite VII semicircular, widely rounded at posterior margin; sternite VIII (Fig. 12a) rounded at posterior margin, disc largely membranous; spiculum ventrale rather long and slender. Abdominal tergite VII semicircular, widely rounded at posterior margin; tergite VIII (Fig. 12b) slightly emarginate at middle of posterior margin, with wide longitudinal membranous area along mid-line. Defensive glands with two large membranous pouches as shown in Fig. 11.

Ovipositor (Fig. 12c and d) strongly sclerotised, gently curved dorsally, with coxites slightly fused; coxite lobe 1 the longest, length ratio of coxite lobes 1–4 about 3:1:2:1; paraproct baculus enlarged at apex. Female genital tubes (Fig. 9b): vagina slender in anterior part, almost gradually inflated in fusiform posterior part; oviduct produced from posterior part of vagina; spermathecal duct short, about 3/4 length of ovipositor, opening at anterior end of vagina; spermatheca globular, at apex of spermathecal duct; bursa copulatrix and spermathecal accessory gland absent.

**Male.** Unknown.

#### Diagnosis

Except for *Falsonannocerusthailandicus* Masumoto, 1986 re-described above and *F.tsuyukii* Masumoto, 1986 from Thailand, *F.haizhuensis* sp. n. is readily differentiated from other congeners by the combination of the following characters: body colour is without colourful metallic lustre; pronotum is subcylindrical, with its length equal to width; intervals of puncture rows on elytra are covered with small tubercles throughout. For the former two species, the new species can be distinguished from them by the following characters:

In *F.thailandicus*, body colour is mostly reddish-brown, dull (Figs 1, 2a); gula is strongly wrinkled (Fig. 3a); antennomere V is wider than long, 1.4 times as wide as long (Fig. 4a); pronotum is covered with dense, well-defined, coarse, deep and subcircular punctures and intervals are distinctly carinate (Fig. 3c); pronotal posterior corners are slightly projected, acute (Fig. 3c); prosternal process with its apex reaching posterior margin of procoxae (Fig. 3e); intervals of puncture rows on elytra are densely covered with small tubercles throughout (Fig. 1a); vagina is rather slender in the anterior part, adruptly inflated in the posterior part (Fig. 9a); spermathecal duct is long, about 1.7 times as long as ovipositor (Fig. 9a). While in *F.haizhuensis* sp. n., body colour is mostly blackish, dull (Fig. 10); gula is almost smooth (Fig. 3b); antennomere V is longer than wide, 1.3 times as long as wide (Fig. 4b); pronotum is covered with dense, ill-defined, coarse, shallow and subcircular punctures and intervals are vaguely carinate (Fig. 3d); pronotal posterior corners are obtusely rounded (Fig. 3d); prosternal process with its apex reaching posterior margin of prothorax (Fig. 3f); intervals of puncture rows on elytra are sparsely covered with small tubercles throughout (Fig. 10a); vagina is slender in the anterior part, almost gradually inflated in the posterior part (Fig. 9b); spermathecal duct is short, about 3/4 length of ovipositor (Fig. 9b).

In *F.tsuyukii*, body colour is mostly reddish-brown, lustrous (Fig. 2b); pronotum is rounder and more convex, 1.2 times as wide as long (Fig. 2b); elytra is widest at the middle (Fig. 2b); intervals of puncture rows on elytra are sparsely scattered with fine tubercles in anterior portion (Fig. 2b). While in *F.haizhuensis* sp. n., body colour is mostly blackish, dull (Fig. 10); pronotum is subcylindrical and less convex, with its length equal to width (Fig. 3d); elytra is widest at the apical 3/7 (Fig. 10a); intervals of puncture rows on elytra are sparsely covered with small tubercles throughout (Fig. 10a).

#### Etymology

The specific epithet is from the Chinese name (Pinyin) of the type locality “Haizhu National Wetland Park”. The name is an adjective.

#### Distribution

China (Guangdong).

## Supplementary Material

XML Treatment for Falsonannocerus thailandicus

XML Treatment for Falsonannocerus haizhuensis

## Figures and Tables

**Figure 2a.**
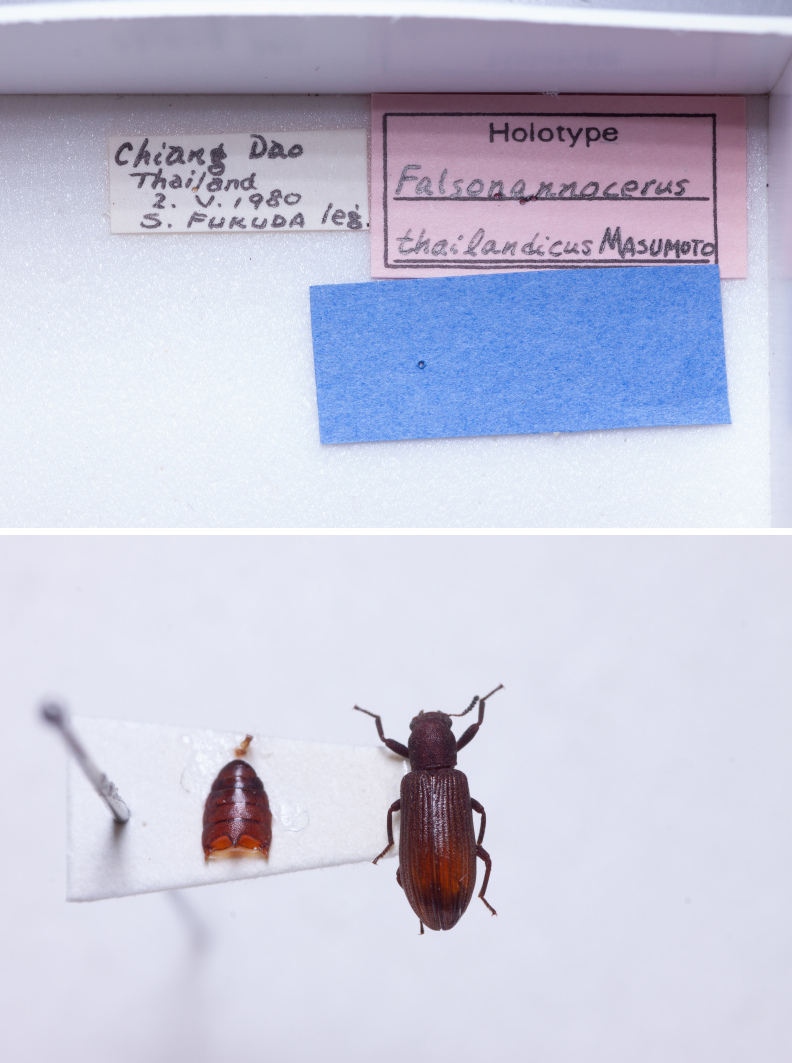
*F.thailandicus* Masumoto, 1986

**Figure 2b.**
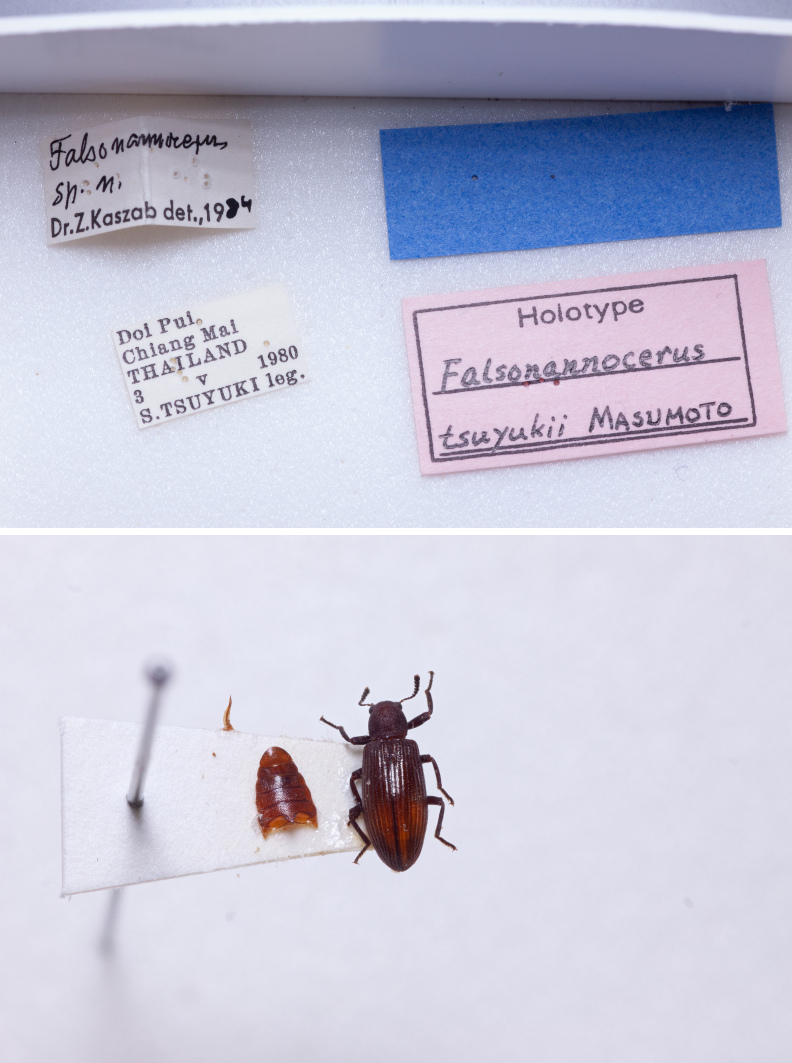
*F.tsuyukii* Masumoto, 1986

**Figure 3a.**
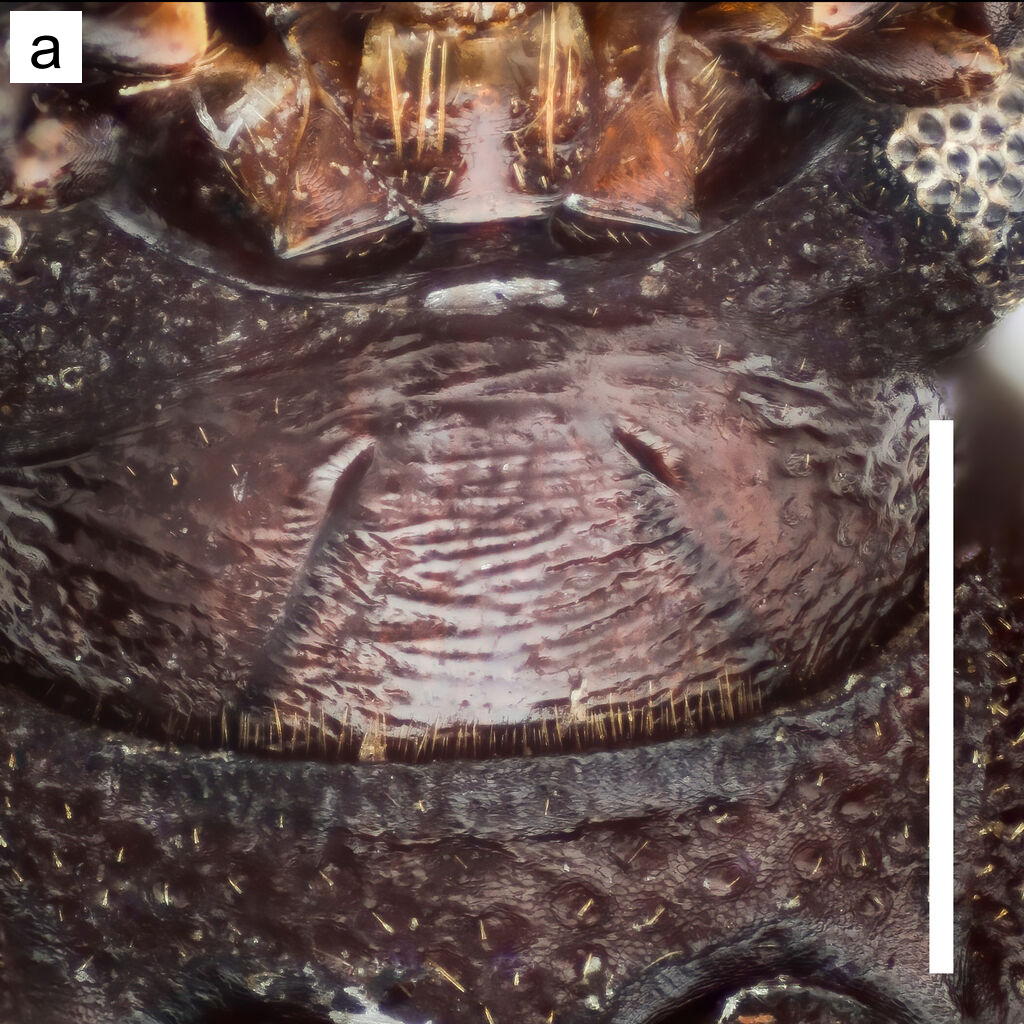
gula of *F.thailandicus* Masumoto, 1986

**Figure 3b.**
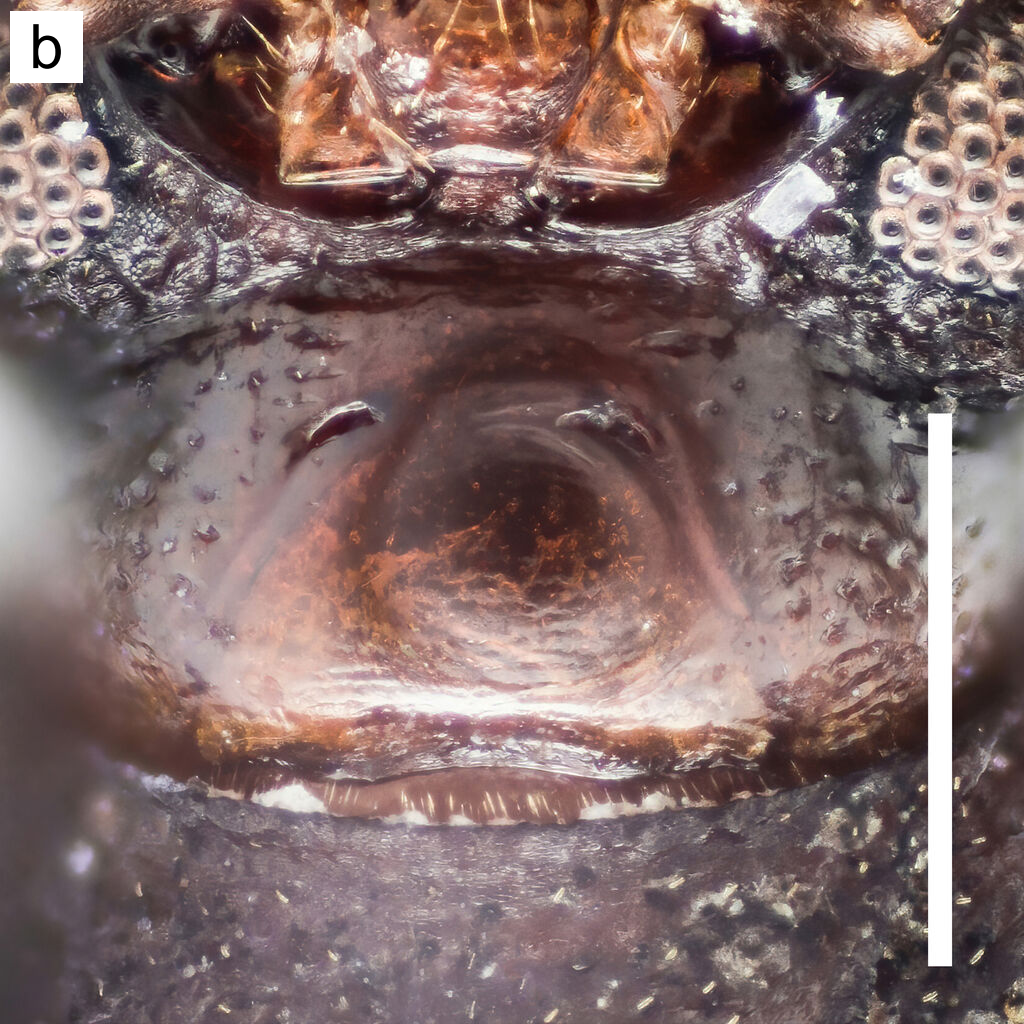
gula of *F.haizhuensis* sp. n.

**Figure 3c.**
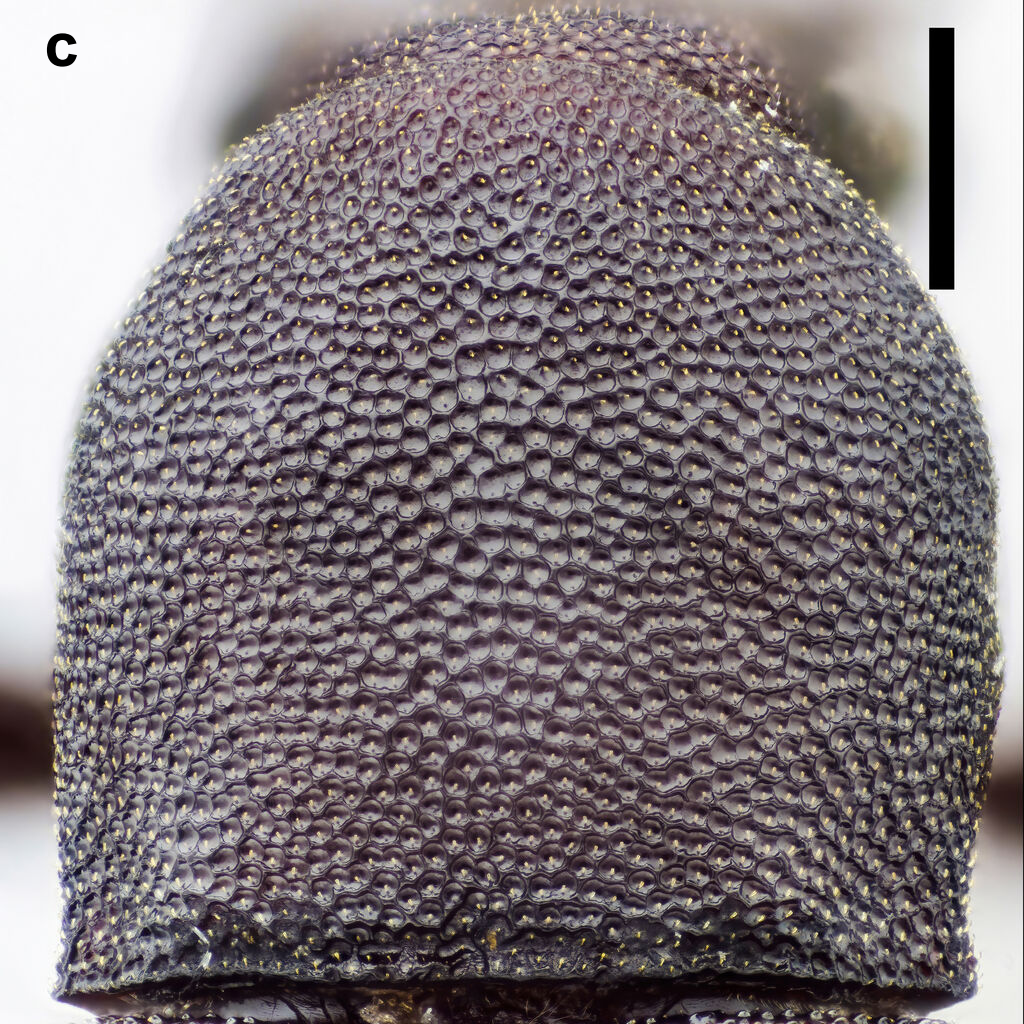
pronotum of *F.thailandicus* Masumoto, 1986

**Figure 3d.**
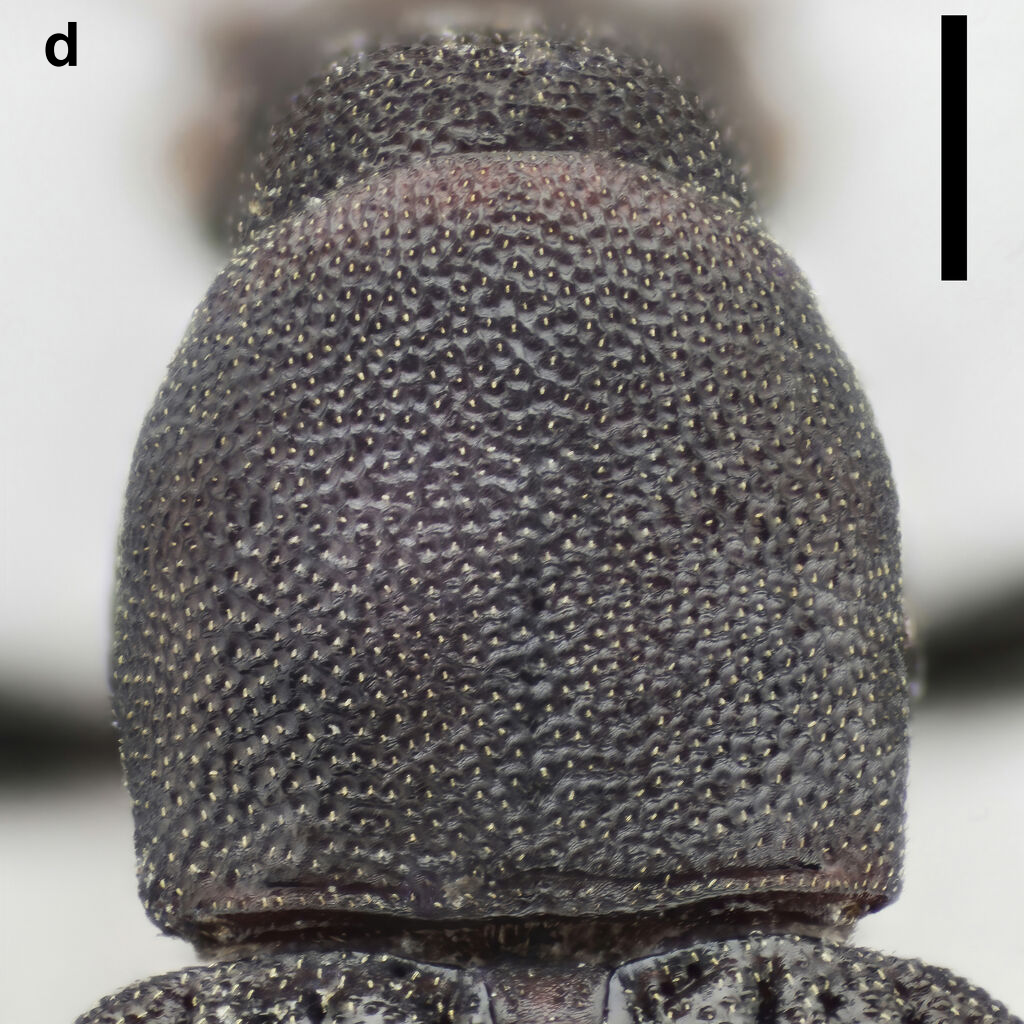
pronotum of *F.haizhuensis* sp. n.

**Figure 3e.**
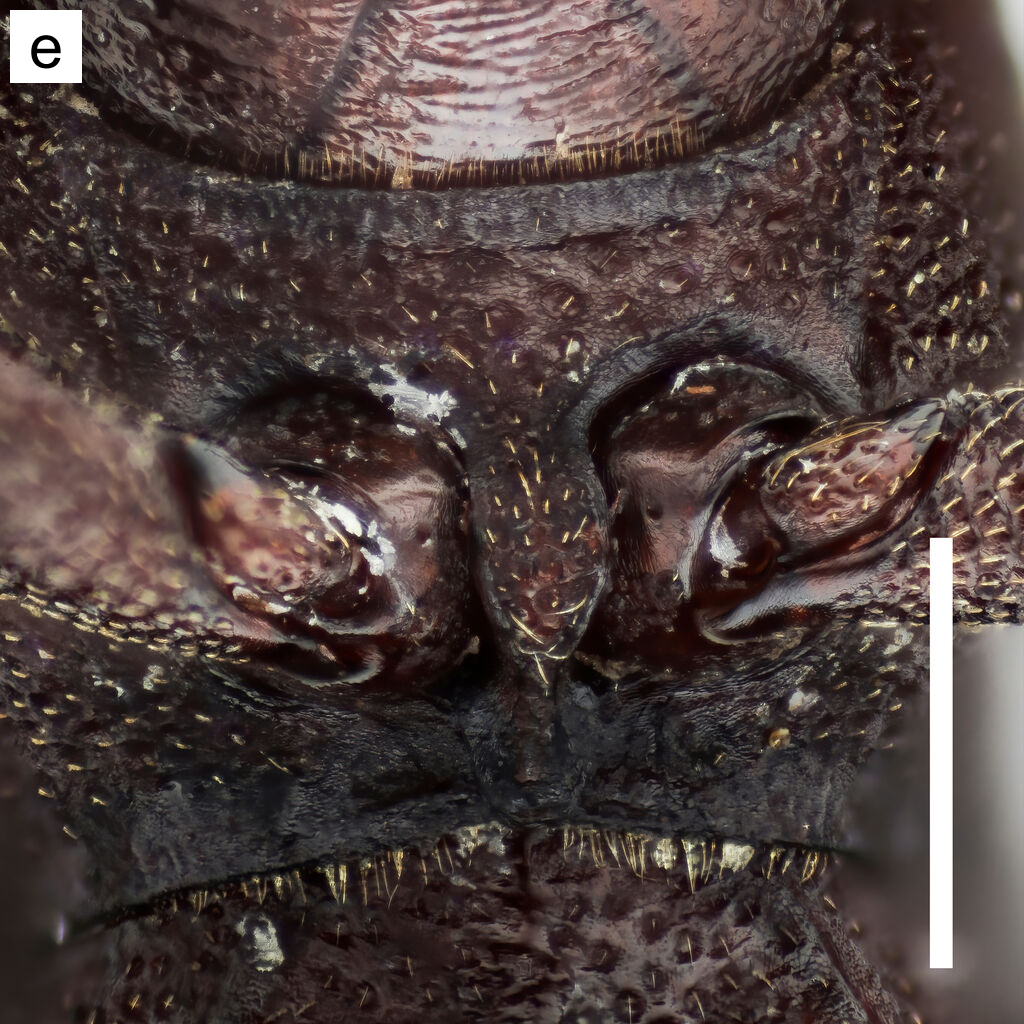
prosternal process of *F.thailandicus* Masumoto, 1986

**Figure 3f.**
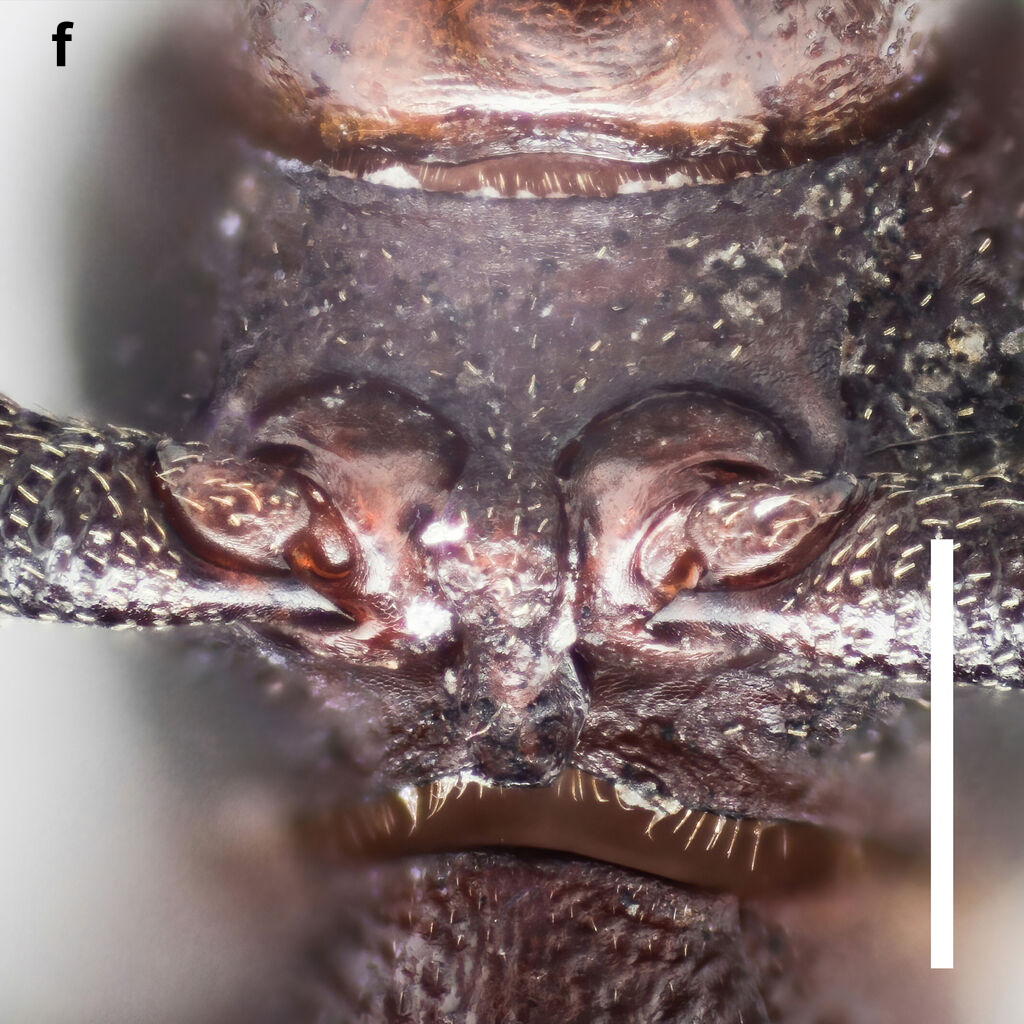
prosternal process of *F.haizhuensis* sp. n.

**Figure 4a.**
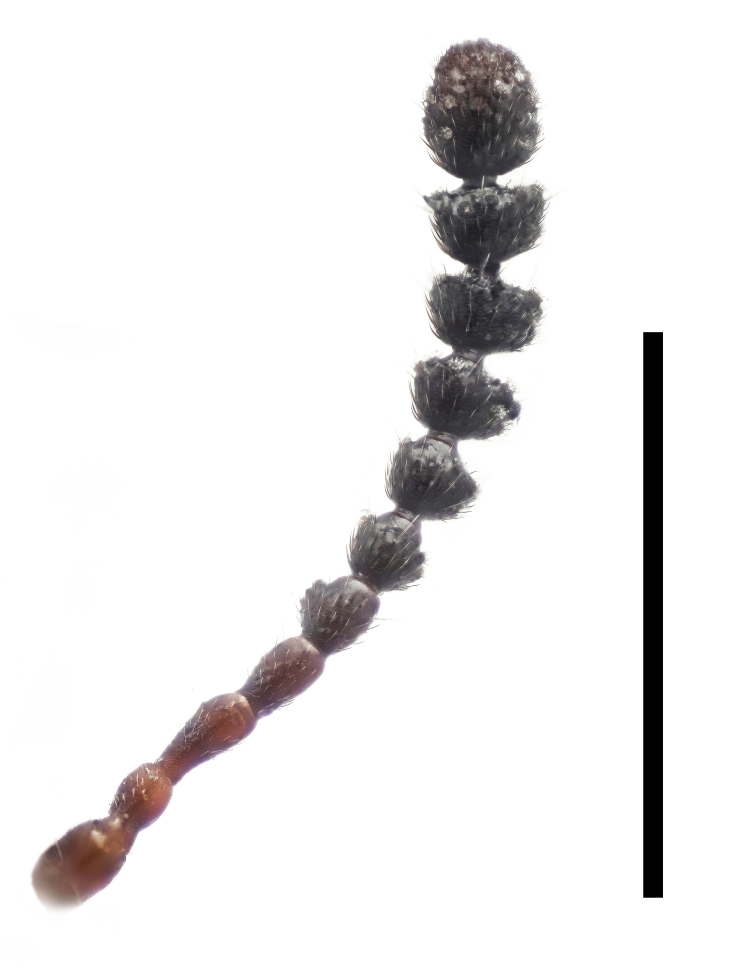
*F.thailandicus* Masumoto, 1986

**Figure 4b.**
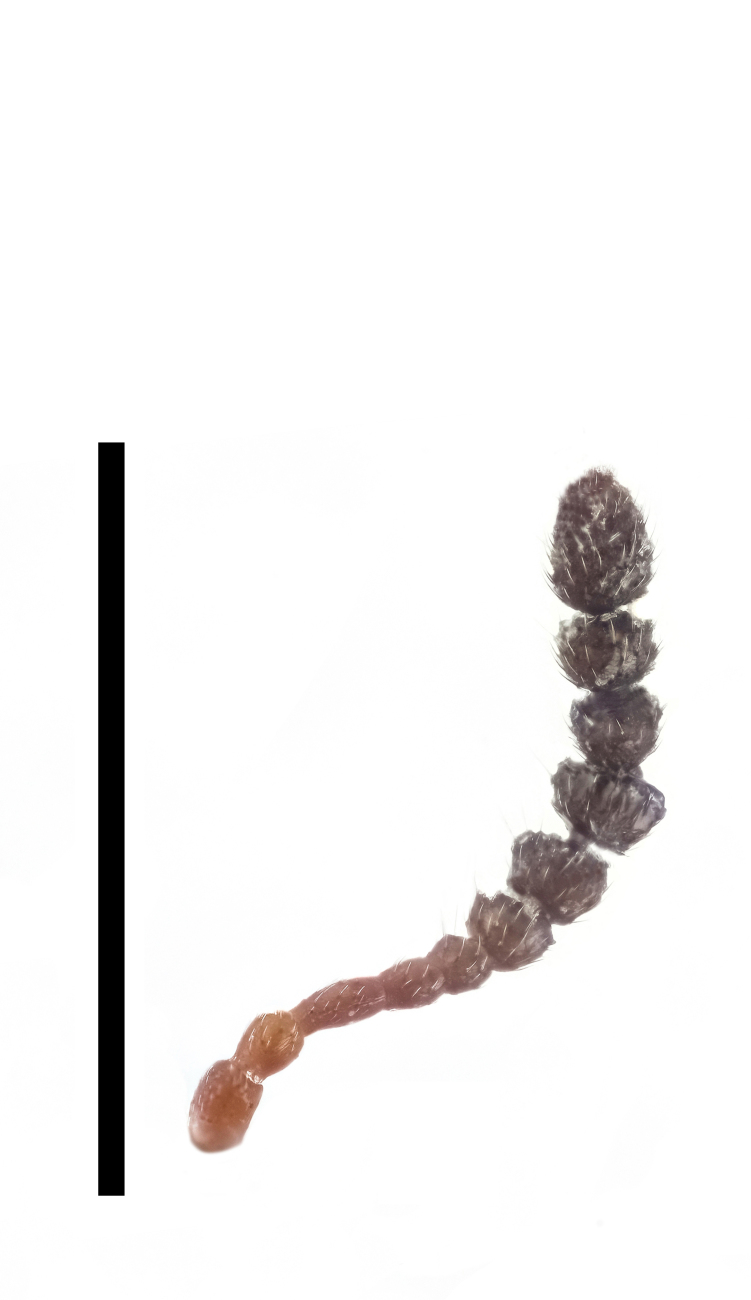
*F.haizhuensis* sp. n.

**Figure 5a.**
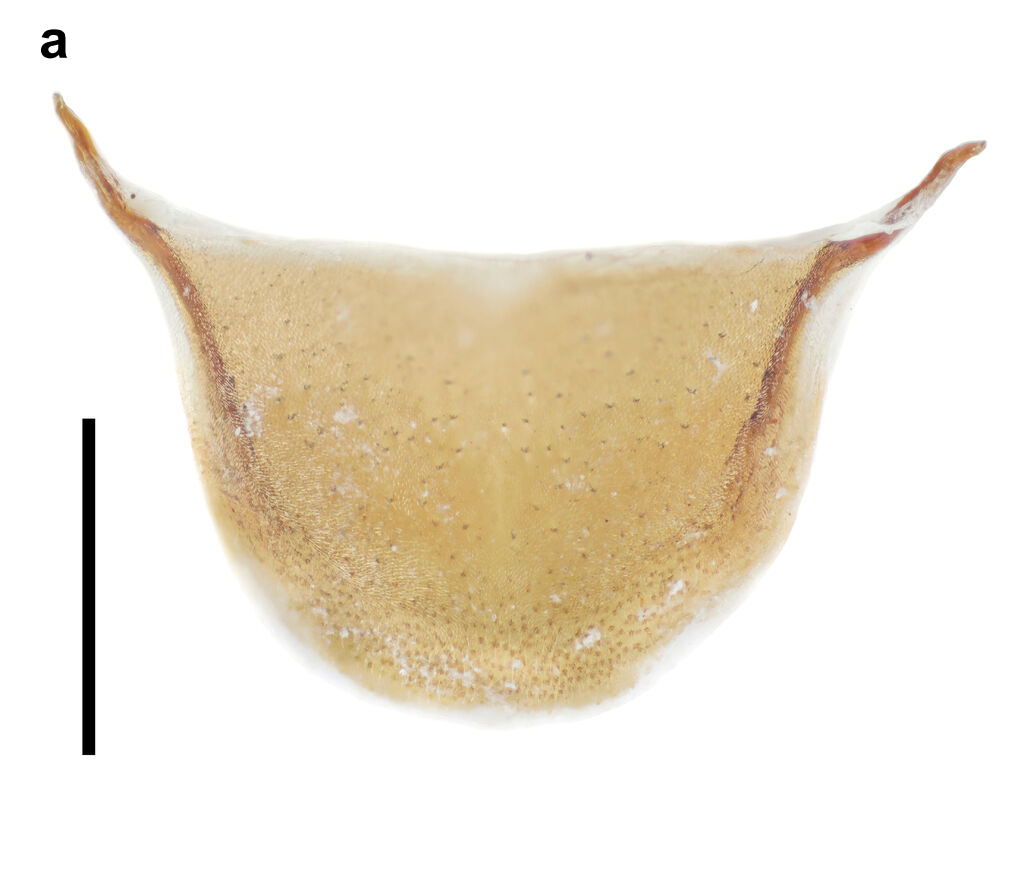
male

**Figure 5b.**
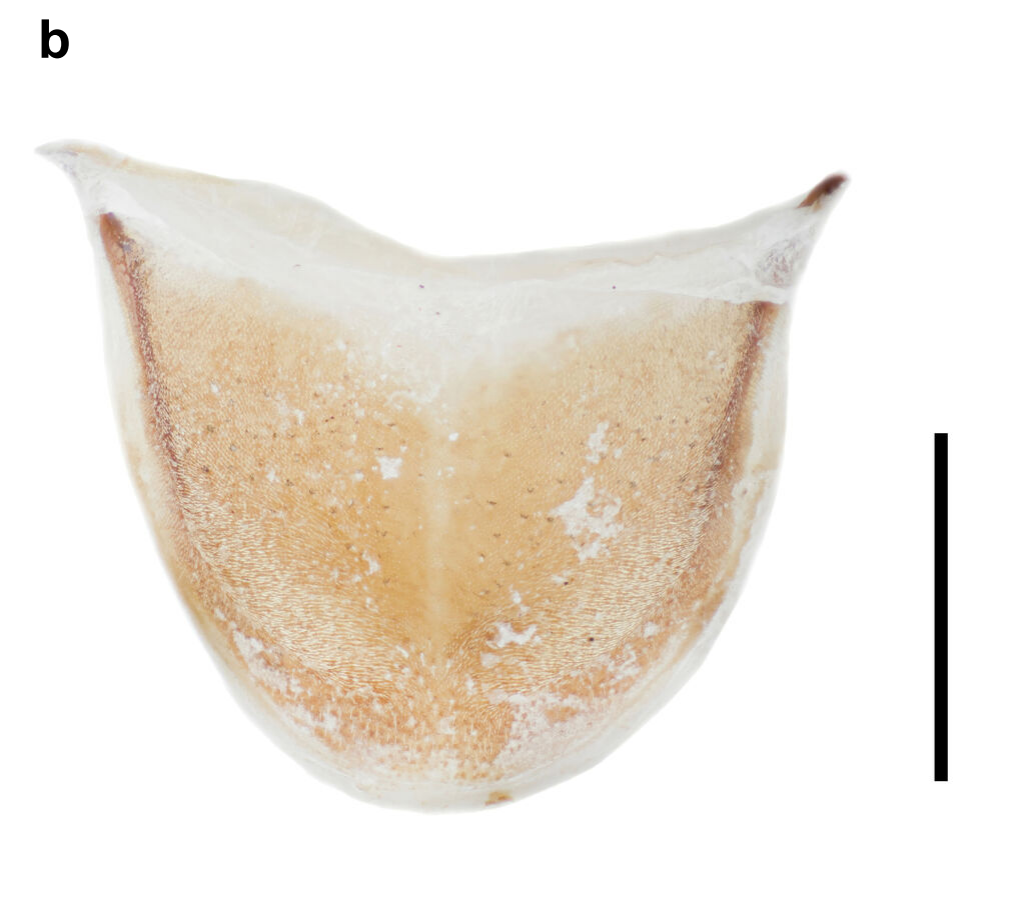
female

**Figure 9a.**
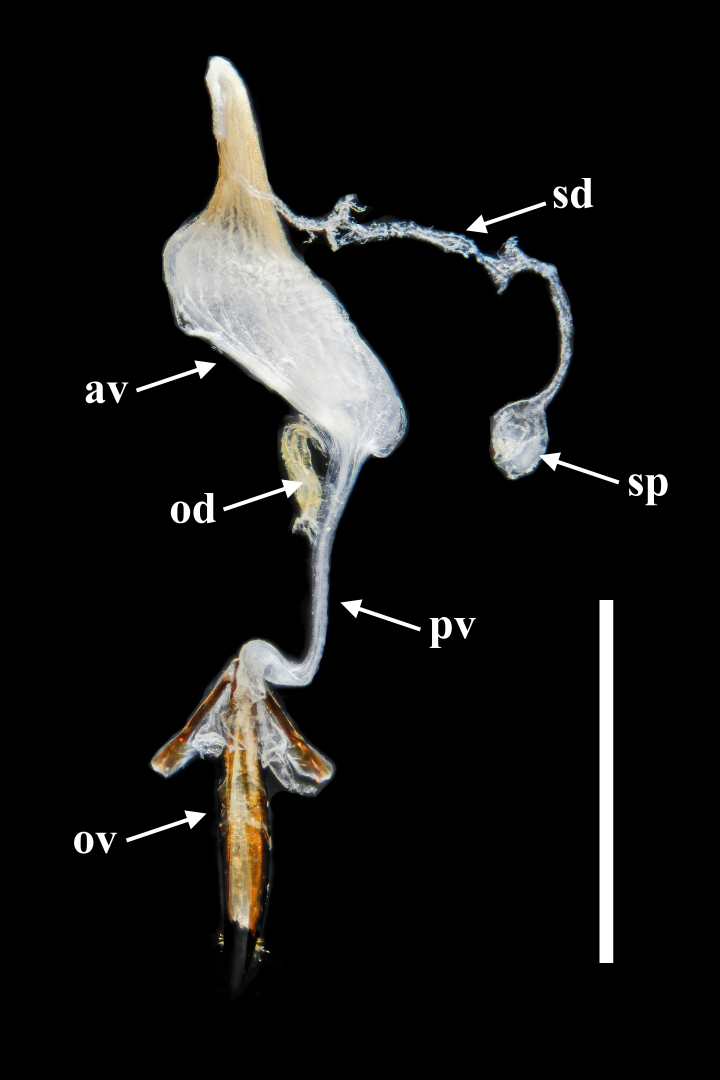
*F.thailandicus* Masumoto, 1986

**Figure 9b.**
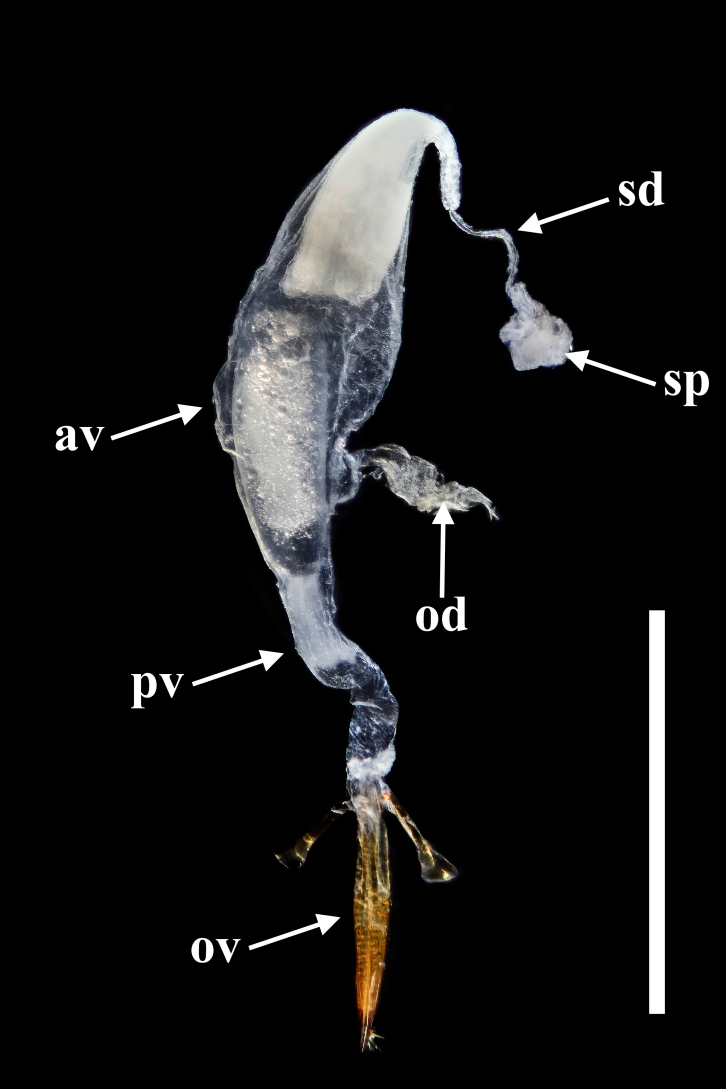
*F.haizhuensis* sp. n.
